# Use of Single Cell Transcriptomic Techniques to Study the Role of High-Risk Human Papillomavirus Infection in Cervical Cancer

**DOI:** 10.3389/fimmu.2022.907599

**Published:** 2022-06-13

**Authors:** Lingzhang Meng, Shengcai Chen, Guiling Shi, Siyuan He, Zechen Wang, Jiajia Shen, Jiajia Wang, Suren Rao Sooranna, Jingjie Zhao, Jian Song

**Affiliations:** ^1^ Institute of Cardiovascular Sciences, Guangxi Academy of Medical Sciences, Nanning, China; ^2^ Center for Systemic Inflammation Research (CSIR), Youjiang Medical University for Nationalities, Baise, China; ^3^ Department of Obstetrics and Gynecology, Affiliated Hospital of Youjiang Medical University for Nationalities, Baise, China; ^4^ Department of Metabolism, Digestion and Reproduction, Imperial College London, Chelsea & Westminster Hospital, London, United Kingdom; ^5^ Life Science and Clinical Research Center, Affiliated Hospital of Youjiang Medical University for Nationalities, Baise, China; ^6^ Department of Radiation Oncology, Renji Hospital, School of Medicine, Shanghai Jiao Tong University, Shanghai, China

**Keywords:** high-risk human papillomavirus, single cell transcriptomic, cervical cancer, tumor infiltrating macrophages, prognosis markers

## Abstract

High-risk human papillomavirus (hrHPV) infection has been associated with a higher probability of progression to cervical cancer. However, several extensive studies have reported that the presence of hrHPV can lead to a better prognosis, but the mechanism of how this occurs is unclear. In this study, microbiological analysis was used to identify HPV infection as a factor for the prognosis of patients with cervical squamous cell carcinoma (CSCC). Comparing the interactions of HPV^+^ and HPV^-^ malignant cells with immune cells as well as the trajectory of malignant cells either with or without HPV, we found that most of the HPV^+^ cells are well differentiated while HPV^-^ cells appear to be hypo-fractionated. Using transcriptomic and immunostaining data, we validated a set of unfavourable molecules in the HPV^-^ CSCC cells, including KRT16, ITGB1, CXCR1, VEGFA, CRCT1 and TNFRSF10B/DR5. This study provides a basis for the development of a rational post-operative follow-up programme and the development of an appropriate treatment plan for patients with cervical cancer.

## Introduction

Persistent high-risk human papillomavirus (hrHPV) infection, particularly the subtypes HPV16 and HPV18 of alpha-HPV, is known to be associated with a higher likelihood of progression to cervical cancer and hrHPV testing has been considered as a routine test for screening for this disease (1). However, several studies using either PCR or sequencing methods to define the HPV infection and its subtypes have reported that mortality rates were significantly lower in the hrHPV^+^ when compared to hrHPV^-^ groups (2, 3). The fact that the presence of hrHPV can lead to a better prognosis in patients has also been observed in oropharyngeal cancer (4). To date the mechanisms involved are not clear and these need to be further explored.

In this study we analysed the clinical parameters of cervical cancer patients as well as their single-cell RNA sequencing (scRNA-seq) data in order to explore the relationships and mechanisms of hrHPV and progression of the cancer. This allowed us to determine the immune extent of cell infiltration as well as the related signaling pathways associated with HPV infection with a view to unravelling relevant potential treatment targets of the disease. This could provide a basis for developing a sound post-operative follow-up programme thus allowing the formulation of appropriate treatment plans for combatting CSCC in the future.

## Materials and Methods

### Collection and Processing of the CSCC RNAseq Dataset

The RNA sequencing dataset and the clinically related data for patients with CSCC originated from the TCGA database (https://portal.gdc.carcinoma.gov/) and consisted of 248 samples. The raw gene expression dataset was processed. Each probe ID received an annotation with respect to the gene from the corresponding platform annotation profile of the GDC website and the raw matrix data received the quantile normalization and log2 conversion. Samples with missing data were excluded. The scRNA-seq data from patients with cervical cancer originated from the gene expression omnibus database and were accessed through NCBI GSE168652 (5). Raw fastq data of scRNA-seq were processed using UMI-tools (6) and viruses present in the single cells were detected using the Viral-Track approach (7). In brief, the sequencing data containing the single cell index were mapped to the virus genome reference database and the status of the single cell was added to the expression matrix so as to correlate with the presence of HPV infection and the corresponding transcriptome. In order to distinguish between benign and malignant cells, inferCNV was used for the analysis of copy number variations (CNVs) in single cell transcriptomes (https://github.com/broadinstitute/inferCNV).

### Building a Microbial Signature

Tumor microbiomes were obtained from the pan cancer microbiome of cBioportal and these were integrated with their respective clinical data (8). The association between the CSCC microbiome and overall survival time in patients from the cancer genome atlas (TCGA) program was studied. Univariate Cox regression analysis was carried out in order to identify the genes associated with survival of individuals (p value < 0.05). Subsequently, the significance of candidate genes was selected using variable importance by using a randomized survival forest (RSF) algorithm. A risk score model with the selected microbial signature was built using multi-variate Cox regression approaches. In addition, the Kaplan-Meier test was employed for a number of gene features and the p-values (log) were determined. Receiver operating characteristic (ROC) analysis was performed for 3- 5- and 10- year overall survival rates and area under the curves (AUCs) were determined for assessing the specificity and sensitivity of the microbial signature.

### Microbiome Analysis

Based on the results generated by sample sequencing of the operational taxonomic unit (OTU), the phyloseq R package was used to calculate the alpha diversity distance matrices. Microbiome analysis was otherwise performed using the http://microbiomeanalyst.ca website. Beta diversity analysis is a comparative analysis between groups of species diversity among different ecosystems or microbial communities and can be used to obtain potential similarities or differences in community composition among differently grouped samples.

### Single Cell RNA Sequencing Analysis

Specific to the integrated analysis that can be obtained from single-cell data, the data from infected new coronavirus and bacterial pneumonia samples as well as non-pneumonia samples were normalized using the SCTransform method (9). These were then analyzed by conducting mutual principal component analysis (PCA). PCA analysis was further conducted for the integrated datasets, and cluster analysis was performed by using uniform manifold approximation and projection (UMAP). The cluster analysis of single-cell data was performed with Seurat’s graph-based clustering method. The resolution of the FindClusters feature was set to 0.1. Subsequently, the clusters were visualized with the UMAP version 0.2.6.0 graph. The R software package Seurat, (version 2.3.4), was subsequently used for data analysis. During quality control, unique molecular modifier (UMI) counts of less than 500 and those with double multiples were removed. Furthermore, cells with >5% of mitochondrial genes and >50% of ribosomal genes were filtered out.

### Functional Assay

The gene features were processed and then analyzed by the method from the WebGestalt webserver for the annotation of involved GO ontology and KEGG pathway (10). Cell-cell communication was analyzed with the CellphoneDB approach and some of the data were illustrated using the InterCellar method (11, 12). Pseudotime analysis was performed using Monocle (version 2.10.1).

### Immunofluorescent Imaging

Biopsies were taken from six cervical patients with cervical cancer. After embedding with a frozen section compound (Leica, #3801480), biopsies were sectioned into 4-μm on a microtome (Leica CM1950). For immunofluorescent staining, the sections were fixed in pre-cooled methanol (-20°C) for 5 minutes, after washing twice with PBS. The sections were then blocked with PBS/5%BSA/Fcγ blocker at 4°C for 1 hour. Primary antibodies were incubated with sections at 4°C overnight. After washing twice with PBS, the sections were incubated with fluorescent-coupled secondary antibodies for 1 hour at room temperature. After two further washes, the sections were mounted and imaged on an immunofluorescence microscope (Leica DMI3000B).

The antibodies/materials used for immunofluorescent imaging included: A488 mouse-anti-human CD206 (Invitrogen, #MA5-23656), PE mouse-anti-human CD163 (Invitrogen, #12-1639-42), A488 mouse-anti-human EpCam (Abcam, #ab237395), mouse-anti-human KRT16/Cytokeratin Pan Antibody Cocktail (Invitrogen, #MA5-13203), rabbit-anti-human CRCT1 (Invitrogen, #PA5-23539), A594 goat-anti-mouse IgG (Invitrogen, #A11032), A594, goat-anti-rabbit IgG (Affinity, #S0006), rabbit-anti-human DR5 (Invitrogen, # PA1-957), A647 donkey-anti-rabbit IgG (Invitrogen, #A32795) and DAPI (Invitrogen, #D21490).

### Statistical Methods

Statistical analyses were carried out using R software (version 3.6.0). Kaplan-Meier tests and ROC analysis were performed as described previously (13, 14). In brief, the “survivor” and “survROC” software packages were utilized for both types of analyses (15). Optimal cut-off data points were calculated using the “survminer” package (16). Single-variate and multi-variate Cox regression correlations were used to assess the prognosis-correlated factors of interest. Hazard ratios and 95% confidence intervals are presented for all the prognosis-correlated factors. Analysis of differences between groups was performed using GraphPad Prism 8.0 software. Student t-test was used for comparison between two groups and P < 0.05 was considered as statistically significant.

## Results

### Microbiome Analysis Grouped Alpha-HPV to Low-Risk CSCC Patients

**Figure 1:**
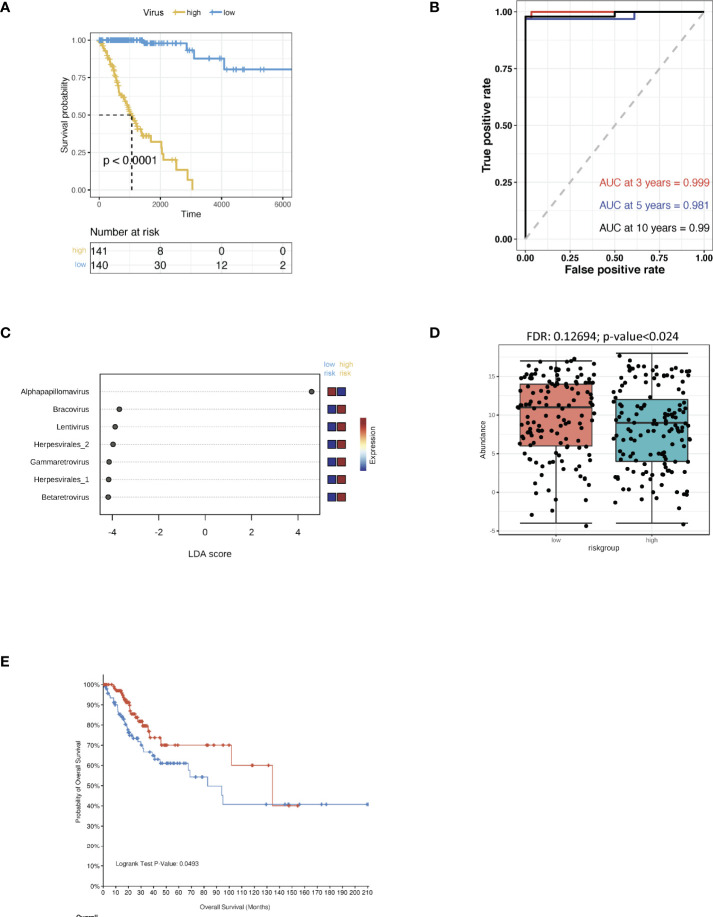
Virus analysis of the prognosis of high and low risk groups of CSCC patients. **(A)** Kaplan-Meier analysis of the risk groups that were defined with prognosis-correlated viruses in the TCGA dataset for CSCC patients. **(B)** Three-, five- and ten-year ROC survival curves of the risk groups for the CSCC TCGA dataset. **(C)** The contribution to the classification is ranked and alpha HPV is observed to support the low-risk group. Linear discriminant analysis was used to study the degree of influence of differential bacteria on sample groupings. **(D)** The abundance of alpha HPV in the low and high risk groups. **(E)** Kaplan-Meier survival analysis of the HPV^+^ group in CSCC patients.

The CSCC microbiomes were obtained from the pan cancer microbiome of cBioportal and then integrated with their respective clinical data. In order to screen for any crucial survival-related viruses, all the viral genomes from the CSCC samples were analysed using multi-variate Cox regression and compared to the TCGA dataset. The CSCC samples were subsequently divided into high and low risk cohorts (**Supporting Data 1**). Kaplan-Meier curves showed that the high-risk group survived for shorter periods when compared to the patients with the low risk viruses (**Figure 1A**). ROC curve analysis of the CSCC cases were then plotted and this showed AUCs of 0.999, 0.981 and 0.99 for 3-, 5- and 10-year survival, respectively (**Figure 1B**). Analysis of the community compositions of both CSCC high and low risk groups showed a similar range of viruses in patients with CSCC at the Class level (**Supplementary Figure 1A**). However, the beta diversity test showed a significant difference in the viruses between high and low risk individuals and those in the high-risk group had significantly lower virus diversity and abundance (**Supplementary Figure 1B**). Correlations between clinical risk and viruses associated with CSCC were investigated based on the Spearman’s correlation test and a significant positive correlation between the low-risk group and alpha-HPV was found (**Supplementary Figures 1C, D**). When correlated with the previous findings, linear discriminant analysis (LDA) classified alpha HPV to the low-risk patients (**Figure 1C**). Also, we found a higher abundance of alpha HPV in the low -risk group compared to that of the high-risk group (**Figure 1D**). The pan cancer microbiome of CSCC data also suggested that the HPV^+^ CSCC patients had a better prognosis compared to the HPV^-^ CSCC patients (**Figure 1E**).

### Transcriptome Profiling of HPV–Infected Cells

**Figure 2:**
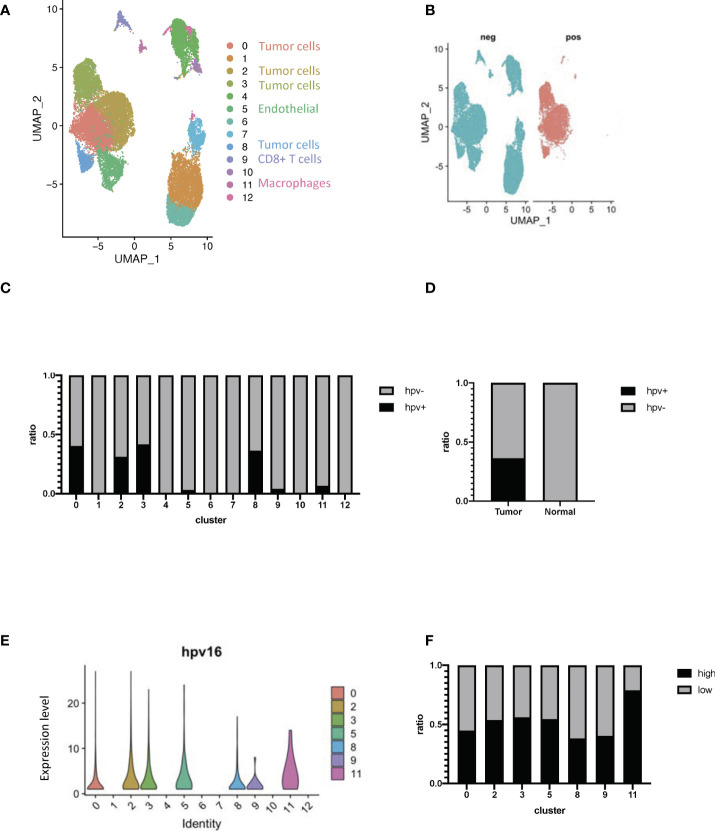
Transcriptome profiling of HPV^+^ cells. **(A)** A UMAP diagram showing the cell clusters **(B)** UMAP diagrams showing HPV^+^ and - cells. **(C)** The bar graph quantifies and compares the proportion of HPV^+^ and ^-^ cells in the main cell types. **(D)** A bar graph to quantify and compare the proportions of HPV^+^ and HPV^-^ cells. **(E)** Violin plots indicating the expression of HPV in the main cell types. **(F)** The bar graph depicts the proportion of HPV high and low cells in the infected cell types. HPV high refers to the cells with hpv16 counts of more than 1.

Cervical cancer scRNA-seq data was used to study HPV infection responses *in vivo* so as to be able to study the transcriptome of different cells within the whole organism. During data mining, human cervical cancer cells were classified into 13 clusters (**Figure 2A**, **Supplementary Figures 2A, B**). HPV-infected cells could be identified by using the raw reads of cervical cancer cells together with the viral track approach (**Figure 2B**, **Supplementary Figure 2C**, **Supporting Data 2**). The infected cells could be separated from any uninfected ones in the main cell types by using the expression levels of HPV (**Figure 2C**). Approximately half of the cells from tumor tissues were found to be infected (**Figure 2D**). As indicated from the analysis, there was a significant level of viral gene expression not only in tumor cells but also in the macrophages and the levels found in the latter were significantly higher than the former (**Figures 2E, F**).

### Identification of Malignant Cells Based on Their CNV Scores

**Figure 3:**
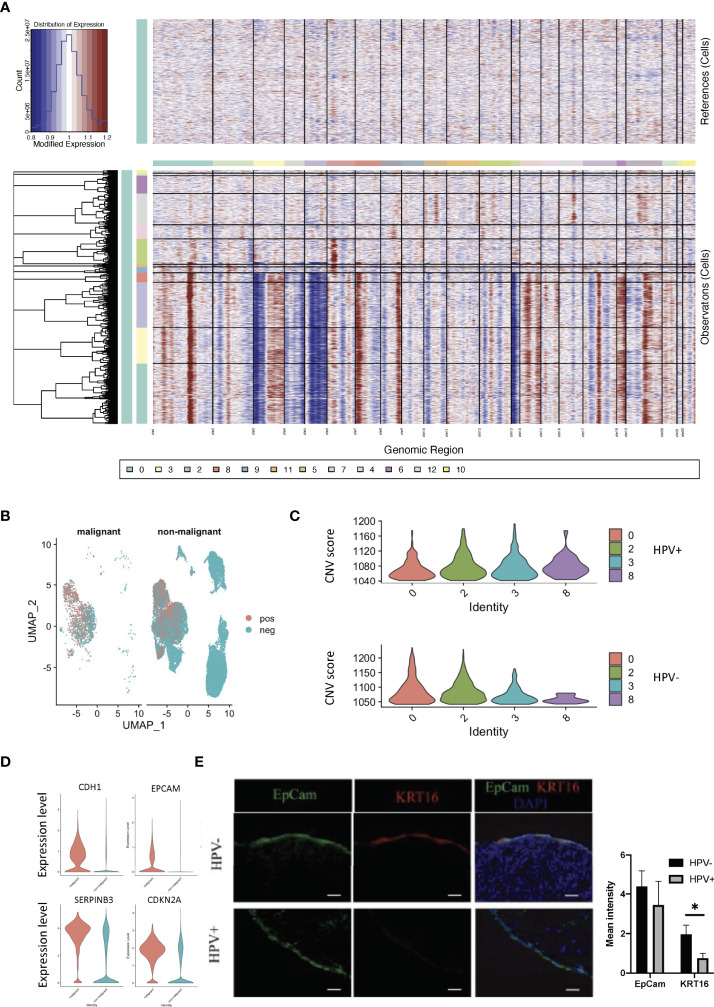
CNV analyses of HPV^+^ tumor cells. **(A)** CNV plots showing the malignant cells in the normal and tumor tissues. **(B)** UMAP diagrams showing non- malignant and malignant cells in the normal and tumor tissues. **(C)** CNV scores in the tumor cell clusters. **(D)** Violin plots indicating the levels of malignant-related gene expression in the non- malignant and malignant cells. **(E)** Representative immunostained photomicrographs of KRT16 in the tissues from HPV^-^ and HPV^+^ patients. EpCam staining refers to the squamous cell carcinoma. Scale bar indicates 50μm. The bar graphs show the mean fluorescent intensity of the indicated staining. * indicates p<0.05 of six samples.

We used infercnv to explore single-cell RNA-seq data from tumours, analysing them for large-scale chromosome copy number alterations (CNA), such as gains or deletions of whole chromosomes or large segments of chromosomes. We used a set of ‘normal’ cells from non-tumour tissues as a reference to analyse the changes in gene expression intensity at various locations on the tumour genome. The relative expression of genes on each chromosome is shown in the form of a heat map, and the tumour genome is either over- or under-expressed when compared to normal cells (**Figure 3A**). Using the infercnv approach, we identified the non-malignant and malignant cells present in the tumor tissue samples, and in these it was found that clusters 0 and 2 in the HPV^-^ cells displayed a higher malignant status when compared to the HPV^+^ cells (**Figures 3B, C**). The CNV scoring were found to be roughly correlated with the most markers of malignancy such as CDH1, EPCAM, CDKN2A and SERPINB3 (**Figure 3D**, **Supplementary Figure 3A**). As CNV scoring may represent the malignant cells more accurately compared with the malignant markers, we used the CNV-defined cells for the further analysis. By doing this, we were able to separate the HPV infected malignant cells from the non- malignant cells and these were then compared to the non-infected malignant cells with respect to the transcriptomic analysis (**Supplementary Figures 3B–E**). We found that keratin 16 was upregulated in most of the HPV^-^ tumor cells. In addition, we validated the gene expression by immunostaining the tissue from HPV^-^ and HPV^+^ patients with both KRT16 and EPCAM antibodies (**Supporting Data 3**, **Figure 3E**).

### Pseudotime Analysis of HPV Infected Tumor Cells

**Figure 4:**
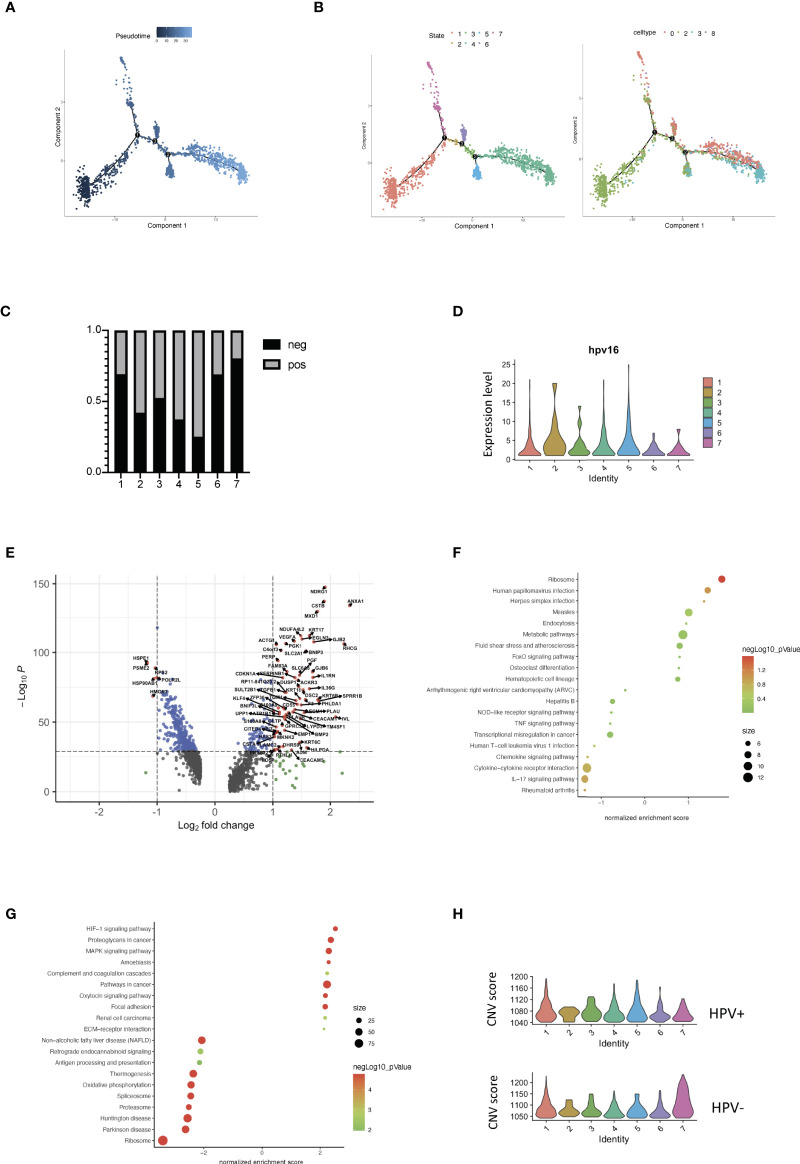
Trajectory analysis of HPV^+^ cervical cancer cells. **(A)** Pseudotime of cervical cancer cells. **(B)** Trajectory analysis of tumor cells in the seven states and in the correlated Seurat clusters (0, 2, 3 and 8). **(C)** The bar chart indicates the ratio of HPV^-^ and HPV^+^ tumor cells in different trajectory states. **(D)** Violin plots showing the expression of HPV16 in the seven states. **(E)** Volcano plots demonstrating the expression patterns and levels of the genes in State 1. **(F)** The enriched KEGG signaling pathway of GSEA analyses for HPV^+^ State 1 cell-correlated gene signatures. **(G)** The enriched KEGG signaling pathway of GSEA analyses for cancer stemness (State 1)-correlated gene signatures. **(H)** CNV scores in different trajectory states.

Each tumor cell could represent a step in time during the development of CSCC. Studying the transcriptome at the single-cell level allows us to identify genes in intermediate states of biological processes, as well as genes in transition states between two different cellular fates. Using the pipeline of Monocle, HPV^+^ and HPV^-^ tumor cells can be sequenced and pseudotimes can be constructed based on the expression trends of the order of genes (**Figures 4A, B**, **Supplementary Figure 4A**, **Supporting Data 4**). 7 trajectory states have been identified and States 1 and 7 refer to the early phase of the pseudotime, and therefore correlate with cancer stemness (**Figures 4A, B**). HPV^-^ tumor cells displayed a high proportion of cancer stemness compared to HPV^+^ cells, while HPV^+^ cells and HPV expression were upregulated in the later states (**Figures 4C, D**). When correlated with the pseudotime states, differential gene expression analysis of State 1 revealed a set of malignant genes such as KRT16 and VEGFA (**Figure 4E**). GSEA functional analyses of DEGs in the HPV^+^ State 1 cells clearly indicated the virus induced signaling and reaction of innate immunity (**Figure 4F**, **Supplementary Figures 4B, D**), while GSEA functional analyses of State 1 correlated with the squamous cell metaplasia and tumorigenesis (**Figure 4G**, **Supplementary Figure 4C**).

Using a branched expression analysis modelling approach, we identified a set of genes that were regulated in a branch-dependent manner based on the trajectory analysis (**Supplementary Figure 4E**), in which CRCT1 was upregulated in the HPV^-^ cells and appeared to play a significantly negative role in the survival prognosis of cervical cancer patients (**Supplementary Figures 4F–H**). In general, the cells in States 1 and 7 displayed a high CNV scores, which were correlated with cancer stemness and progression. Compared to HPV^-^ cells, HPV^+^ malignant cells were enriched in the intermediate phases, suggesting a crucial role of HPV infection in the progression of cervical cancer cells (**Figure 4H**).

### Cell-Cell Communication Between Immune and HPV^-^Infected Tumor Cells

**Figure 5:**
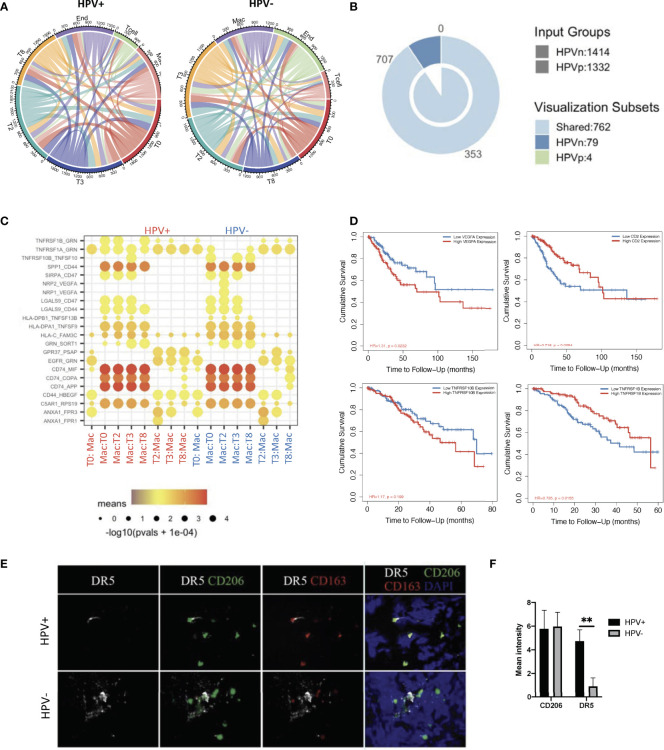
Cell-cell communication between immune cells and HPV^+^ tumor cells. **(A)** A network of interactions between immune and either HPV^+^ or HPV^-^ tumor cells. The lines represent the pointing relationships. **(B)** A Venn diagram showing the shared and unique cell-cell interactions of immune cells with HPV^+^ or HPV^-^ tumor cells. **(C)** Dot plots showing the most significant interactions (mean>1) of macrophages with either HPV^+^ or HPV^-^ tumor cells and the significance of their relationships. The horizontal coordinates are cell type interactions and the vertical coordinates are protein interactions, with the larger dots indicating smaller p-values and the colours representing the average expression. **(D)** Kaplan-Meier survival analysis of VEGFA, CD2, TNFRSF10B and TNFRSF1B in the CSCC patients. Representative immunostained photomicrographs of TNFRSF10B/DR5 **(E)** on the biopsied sections from HPV^+^ or HPV^-^ patients. CD206 and CD163 staining refer to the tumor infiltrated macrophages. Scale bar indicates 15μm. **(F)** Bar graphs showing the quantification of TNFRSF10B DR5 and CD206 respectively. ** indicate p<0.01 of six samples.

In order to investigate the interactions of HPV^+^ and HPV^-^ cells with immune cells in the tumor microenvironment, where tumor cells develop and immune cells interact very closely with them, we constructed an intercellular communication network by using the cellphoneDB method (12). The results showed that endothelial cells and macrophages had much more interactions with tumour cells when compared to T cells (**Figure 5A**, **Supporting Data 5**). Specifically, HPV^-^ tumor cells displayed more unique cell-cell interactions when compared to HPV^+^ cells, and this is mainly due to the presence of the intergrin b1-extracellular matrix complex (**Figure 5B**, **Supporting Data 5**).

Of those unique cell-cell interactions of HPV^-^ cells, ITGB1 and CXCR1 were found to be associated with unfavourable prognosis of CSCC (**Figure 5B**, **Supplementary Figures 5A, B**). In particular, tumour cell type 0 appeared to have a significantly higher amount of cellular communication with macrophages, which correlated with the high proportion of tumor infiltrated macrophages in CSCC (Figure 5A, **Supplementary Figure 5C**). Also, it was found that there was a significantly enhanced VEGFA and TNFRSF10B/DR5 signaling association with macrophages interacting with HPV^-^ types 0 and 2 tumor cells, which was confirmed by tissue staining and appeared to play a significantly negative role in the prognosis of cervical cancer patients (**Figures 5C–F**). On the other hand, HPV^+^ tumor cells upregulated the favourable gene TNFRSF1B derived pathway (**Figures 5C, D**). Also, T cells interacting with various HPV^+^ of tumor cells had significantly upregulated CD2 signalling pathways (**Figure 5C**, **Supplementary Figure 5D**), while both HPV^+^ and – cells showed similar interactions with endothelial cells (**Supplementary Figure 5E**). Taken together, these data indicate a distinct scenario of HPV^+^ and HPV^-^ tumor cells in cervical cancer, which might explain the observation that mortality rates were lower in the hrHPV^+^ when compared to hrHPV^-^ CSCC patients.

## Discussion

**Figure 6:**
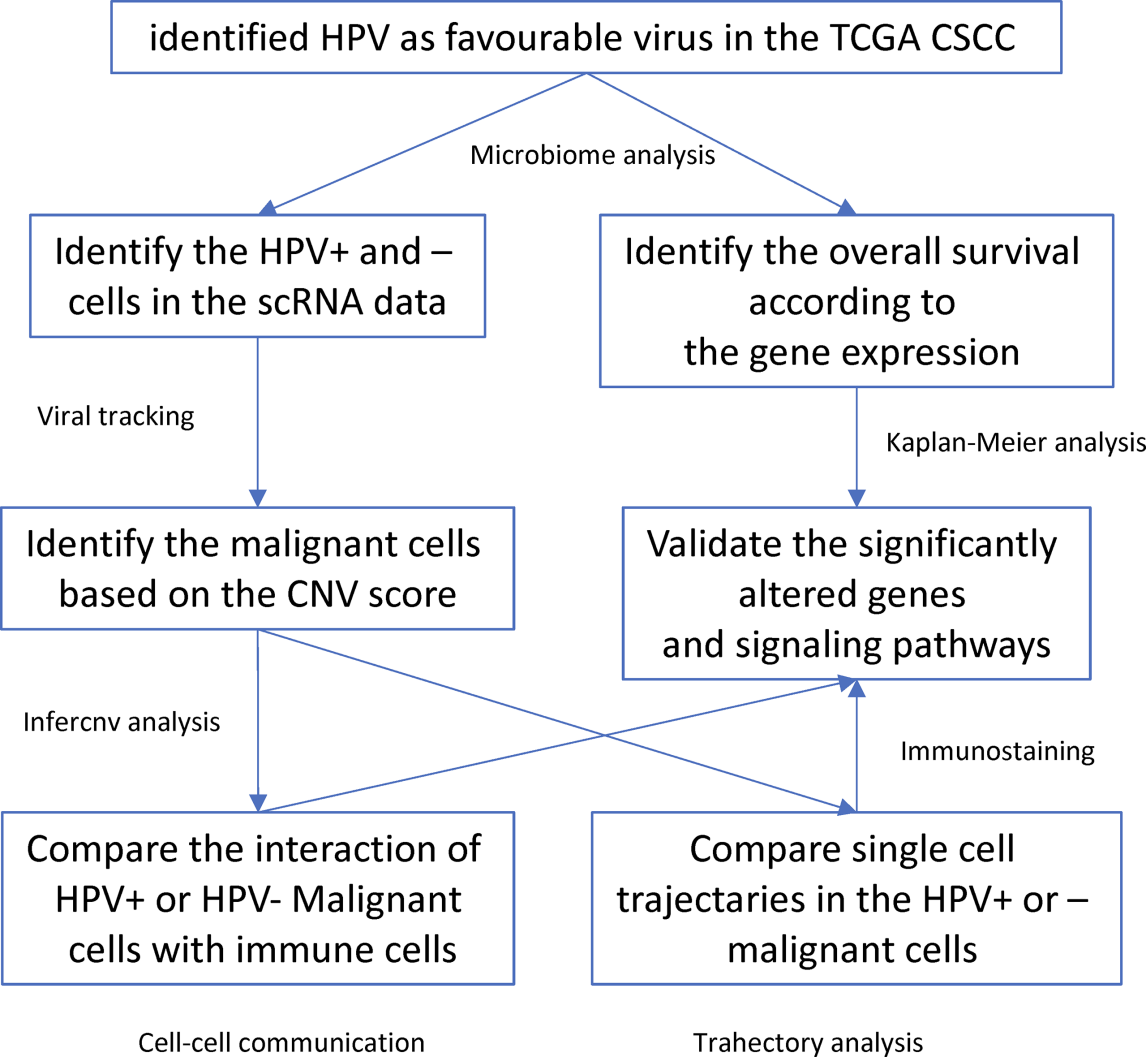
Workflow diagram of the current study.

HPV infection is a critical factor involved in the etiology and progression of cervical cancer. In this study, we used microbiological analysis to identify HPV as a favourable virus for the prognosis of CSCC patients. In addition, we used scRNA data and viral tracking methods to identify HPV infections and their subtypes. Then we identified malignant cells which together with the viral tracking methods allowed identification of DEGs by comparing HPV^+^ and HPV^-^ malignant cells. We also built a trajectory analysis for both HPV^+^ or HPV^-^ cells and identified the interactions between immune and malignant cells (**Figure 6**). We found that HPV^+^ cells upregulated the favourable gene-derived signaling, including CD2 and TNFRSF1B, whereas HPV^-^ cells upregulated the signaling that was derived by the CSCC unfavourable molecules such as ITGB1, CXCR1, VEGFA and TNFRSF10B/DR5. Monoclonal antibody targeting of these molecules, such as Bevacizumab and Tigatuzumab (17, 18), have been developed and are currently under clinical trials for cancer patients other than CSCC, which suggests that they may be beneficial to the HPV^-^ CSCC patients in accordance with our study.

The fact that hrHPV negativity is associated with a good prognosis for tumors does not necessarily mean that hrHPV is not involved in the etiology of cancer development as the HPV^-^ cases may have been infected with the virus at an earlier time-point before the cancer was diagnosed. HrHPV negativity is usually associated with advanced tumors, suggesting that these viruses may become undetectable at later stages of the carcinogenic process (19). Our data would suggest that hrHPV^-^ cells in tumor tissues are likely to indicate a loss of hrHPV expression in a subset of cells during carcinogenesis. However, the mechanism whereby this loss occurs is not fully understood. It is possible that hrHPV^-^ tumor cells may lose their internal mutational control and thus acquire genetic mutations associated with malignant growth and proliferation potential. In this scenario, the hrHPV^+^ tumor cells may be under better control of the viral protein by the immune system due to their expression and, therefore, this results in a relatively more positive prognosis of CSCC in these patients. In the present study we identified DEGs of hrHPV^+^ and hrHPV^-^ tumor cells in different clusters and states, as well as differential cellular communication and interactions of these cells with immune cells. However, due to limitations in the sequencing depth of scRNA-seq, we were unable to investigate possible gene mutations in hrHPV^-^ tumor cells. In the future, we hope to remedy this failing by increasing the sequencing depth of single-cell sequencing with a view to obtaining a more comprehensive comparison between hrHPV^+^ and hrHPV^-^ tumor cells.

HPV infection is a major cause of cervical cancer and this study focuses on the correlation between HPV positivity and prognosis. In contrast to the single cause of cervical cancer, HNSCC is a cancer that arises as a result of exposure to carcinogens (e.g. alcohol and/or tobacco) or through malignant transformation due to HPV infection. Indeed, HPV^-^associated HNSCC has been found to exhibit unique biological and clinically relevant features, and the presence of the virus provides a survival advantage compared to its absence. However, because of the large number of cases of HPV^-^unrelated tumours, it cannot be ruled out that the difference in prognosis between HPV^+^ and HPV^-^ cases is due to the fact that they have distinct etiologies, which differ in terms of pathogenesis. Therefore, a long-term follow-up of the presence of HPV in HNSCC patients is needed to establish the relevance of HPV changes in the disease.

In this study, the clinical and single-cell sequencing data of cervical cancer patients were analyzed in order to explore the relationships and mechanisms of hrHPV and cervical cancer progression. Our current data suggest that HPV^-^ cervical cancer cells can exhibit more cancer stemness properties, both with respect to analysis of the gene expression profile characteristics of the cancer and experimental validation. We also found that regulatory programs controlling stemness function are active in cancer, and continued research in this area will contribute to a better understanding of the mechanisms of the progression of HPV^-^ cervical cancer and help to combat treatment resistance in cancer patients.

The current study yielded information regarding the immune cell interactions and the related signaling pathways associated with HPV infection in cervical cancer patients. It is hoped that this will reveal potential targets for treatment regimens for CSCC patients. In addition, it may provide a basis for the development of a useful post-operative follow-up programme that will benefit the prognosis of future patients with CSCC.

## Data Availability Statement

The datasets presented in this study can be found in online repositories. The names of the repository/repositories and accession number(s) can be found in the article/**Supplementary Material.**

## Ethics Statement

The studies involving human participants were reviewed and approved by Ethical Committee of Youjiang Medical University for Nationalities. The patients/participants provided their written informed consent to participate in this study.

## Author Contributions

JS and JZ designed this study and performed the data analysis. LM, SC and GS performed partial data analysis and performed immunofluorescent imaging for this study. SC and GS performed clinical diagnosis and surgically isolated biopsies for this study. SH, ZW, JJS and JW performed partial data analysis for this study. SS helped to compose and review the manuscript for this study. LM, SC and GS contributed equally to this study. All authors contributed to the article and approved the submitted version.

## Funding

This study was funded by grants from the National Science Foundation of China (#31970745), Guangxi Natural Science Foundation (#2020GXNSFAA259050), Youjiang Medical University for Nationalities (#yy2019bsky001), and Baise Development of Science & Technology Research Program(#20170505).

## Conflict of Interest

The reviewer JL declared a shared affiliation with the author JS to the handling editor at time of review.

The remaining authors declare that this research was conducted in the absence of any commercial or financial relationships that could be construed as a potential conflict of interest.

## Publisher’s Note

All claims expressed in this article are solely those of the authors and do not necessarily represent those of their affiliated organizations, or those of the publisher, the editors and the reviewers. Any product that may be evaluated in this article, or claim that may be made by its manufacturer, is not guaranteed or endorsed by the publisher.

## Supplementary Material

Supplementary Figure 1 Virus analysis of the prognosis of high and low risk groups of CSCC patients. **(A)** Composition of the CSCC viruses at the class level in patients with high and low risks. **(B)** A two-dimensional scatter plot of the non-metric multidimensional scale analysis of virus class levels in CSCC cancer patients. **(C)** Heatmap cluster analysis of the viruses in the high and low risk groups of CSCC patients. **(D)** The correlation coefficients which correlated with Herpesvirus.

Supplementary Figure 2 Virus analysis of the high and low risk groups of CSCC prognosis. **(A)** Violin plots indicating the expression and distribution of marker genes. **(B)** A UMAP diagram showing the cell clusters in normal and tumor tissues in the CSCC patients **(C)** A bar graph showing the mapped reads of HPV in the CSCC scRNA data. **(D)** UMAP diagrams showing high and low dose of HPV expressing cells.

Supplementary Figure 3 Gene expression analyses of HPV infected tumor cells. **(A)** Feature plots showing the malignance related genes in the non- malignant and malignant cells. **(B–E)** Volcano plots demonstrating the expression patterns and levels of the genes in non-malignant and malignant cell types.

Supplementary Figure 4 Trajectory analysis of HPV infected cervical cancer cells. **(A)** Trajectory analysis of HPV^-^ (n) and HPV^+^ (p) tumor cells in different states. States 1 and 7 refer to the beginning and the end of the development. **(B)** Volcano plots demonstrating the expression patterns and levels of the genes in the HPV^+^ cells of State 1. **(C)** The enriched gene ontology of GSEA GO analysis for cancer stemness (State 1)-correlated gene signatures. **(D)** The enriched gene ontology of GSEA GO analysis for HPV^+^ State1 cell-correlated gene signatures. **(E)** BEAM (Branched expression analysis modelling) was used to find the genes that were regulated in a branch-dependent manner. A heatmap showing the temporal differential genes with the top 100 extracted. The horizontal axis of the heatmap refers to the proposed time. The genes shown are those that are highly expressed in the indicated clusters at the focus of the proposed time sort. **(F)** A dot plot showing the expression of CRCT1 in the seven states of HPV^+^ or HPV^-^ tumor cells. **(G)** Kaplan-Meier analysis of overall survival according to the gene expression of CRCT1 in CSCC patients from the TCGA dataset. **(H)** Representative immunostained photomicrographs of CRCT1 in the tissues obtained from HPV^-^ and HPV^+^ patients. EpCam staining refers to the squamous cell carcinoma. Scale bar indicates 50μm. Bar graphs depicted the mean fluorescent intensity of the indicated staining. * indicates p<0.05 of six samples.

Supplementary Figure 5 Cell-cell communication between immune cells and HPV^-^infected tumor cells.Kaplan-Meier survival analysis of the HPV^-^ tumor cells using the interaction genes **(A)** ITGB1 and **(B)** CXCR1 in the CSCC patients. **(C)** A bar graph showing the CIBERSORT estimated infiltration of immune cell subsets of samples from HPV^+^ and HPV^-^ CSCC patients. Dot plots showing the most significant interactions (mean>1) of endothelial cells **(D)** and T cells **(E)** with either HPV^+^ or HPV^-^ tumor cells and the significance of their relationships. The horizontal coordinates are cell type interactions and the vertical coordinates are protein interactions, with the larger dots indicating smaller p-values and the colours representing the average expression.

## Abbreviations

hrHPV, High-risk human papillomavirus; CSCC, Cervical squamous cell carcinoma; scRNA-seq, Single-cell RNA sequencing; CNVs, Copy number variations; UMAP, Uniform manifold approximation and projection; ROC, Receiver operating characteristic; PCA, Principal component analysis.
