# Supplementary figures and images for: Use of Single Cell Transcriptomic Techniques to Study the Role of High-Risk Human Papillomavirus Infection in Cervical Cancer
Source: Front Immunol. 2022 Jun 13;13:907599. doi: 10.3389/fimmu.2022.907599 (PMC9236134; doi:10.3389/fimmu.2022.907599)

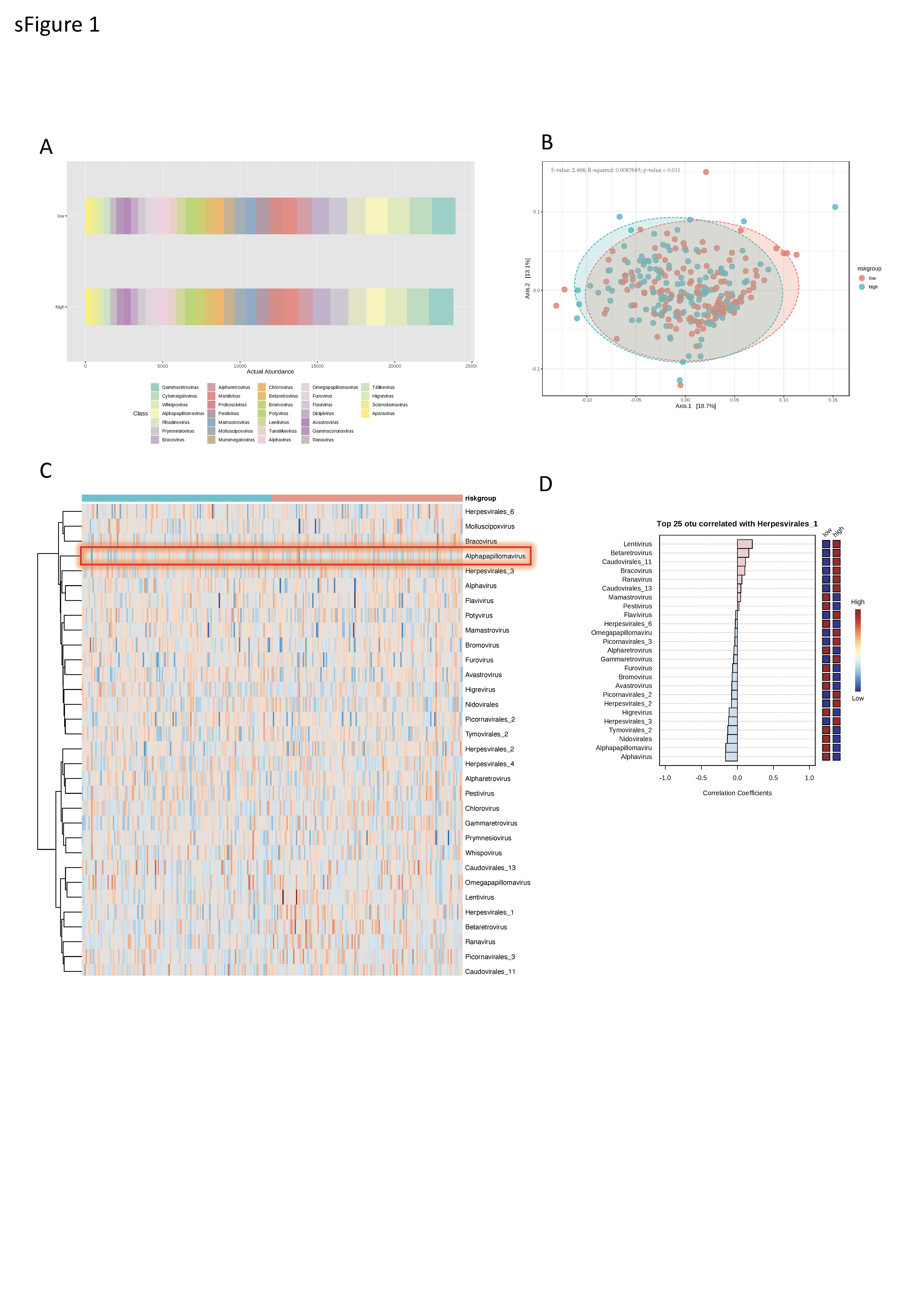

Supplement: Supplementary Figure 1 — Virus analysis of the prognosis of high and low risk groups of CSCC patients. (A) Composition of the CSCC viruses at the class level in patients with high and low risks. (B) A two-dimensional scatter plot of the non-metric multidimensional scale analysis of virus class levels in CSCC cancer patients. (C) Heatmap cluster analysis of the viruses in the high and low risk groups of CSCC patients. (D) The correlation coefficients which correlated with Herpesvirus. [file Image_1.tiff]

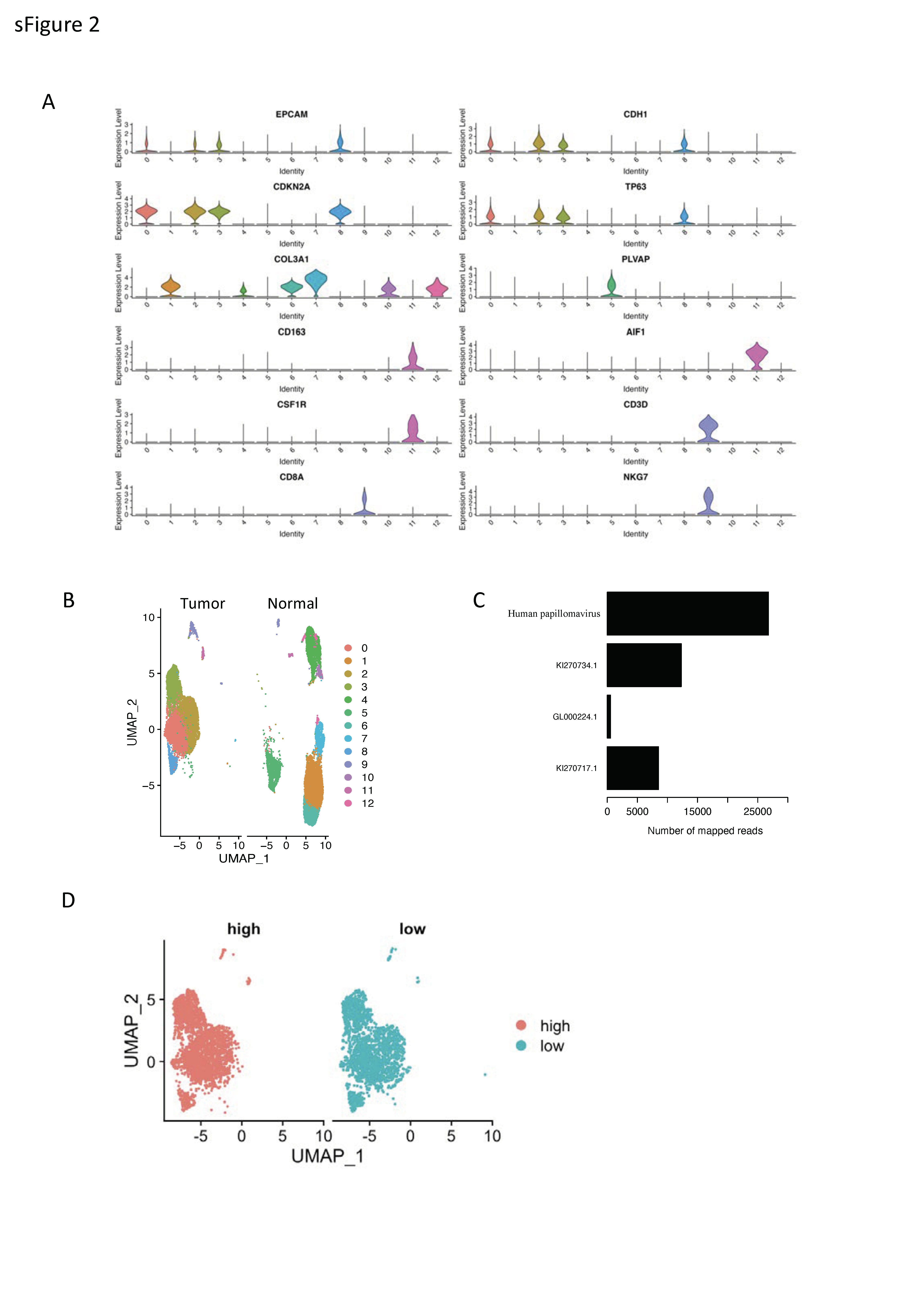

Supplement: Supplementary Figure 2 — Virus analysis of the high and low risk groups of CSCC prognosis. (A) Violin plots indicating the expression and distribution of marker genes. (B) A UMAP diagram showing the cell clusters in normal and tumor tissues in the CSCC patients (C) A bar graph showing the mapped reads of HPV in the CSCC scRNA data. (D) UMAP diagrams showing high and low dose of HPV expressing cells. [file Image_2.tiff]

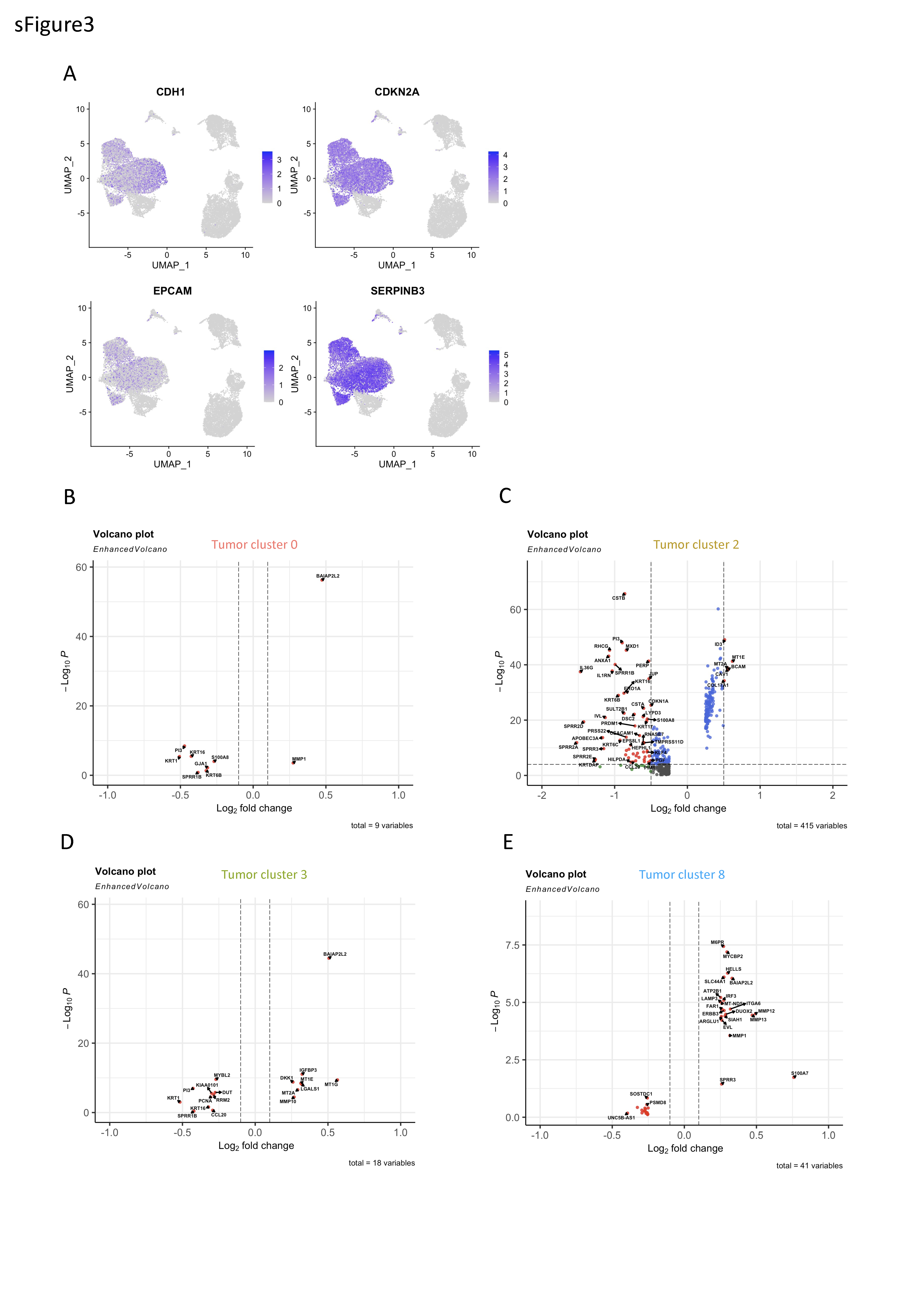

Supplement: Supplementary Figure 3 — Gene expression analyses of HPV infected tumor cells. (A) Feature plots showing the malignance related genes in the non- malignant and malignant cells. (B–E) Volcano plots demonstrating the expression patterns and levels of the genes in non-malignant and malignant cell types. [file Image_3.tiff]

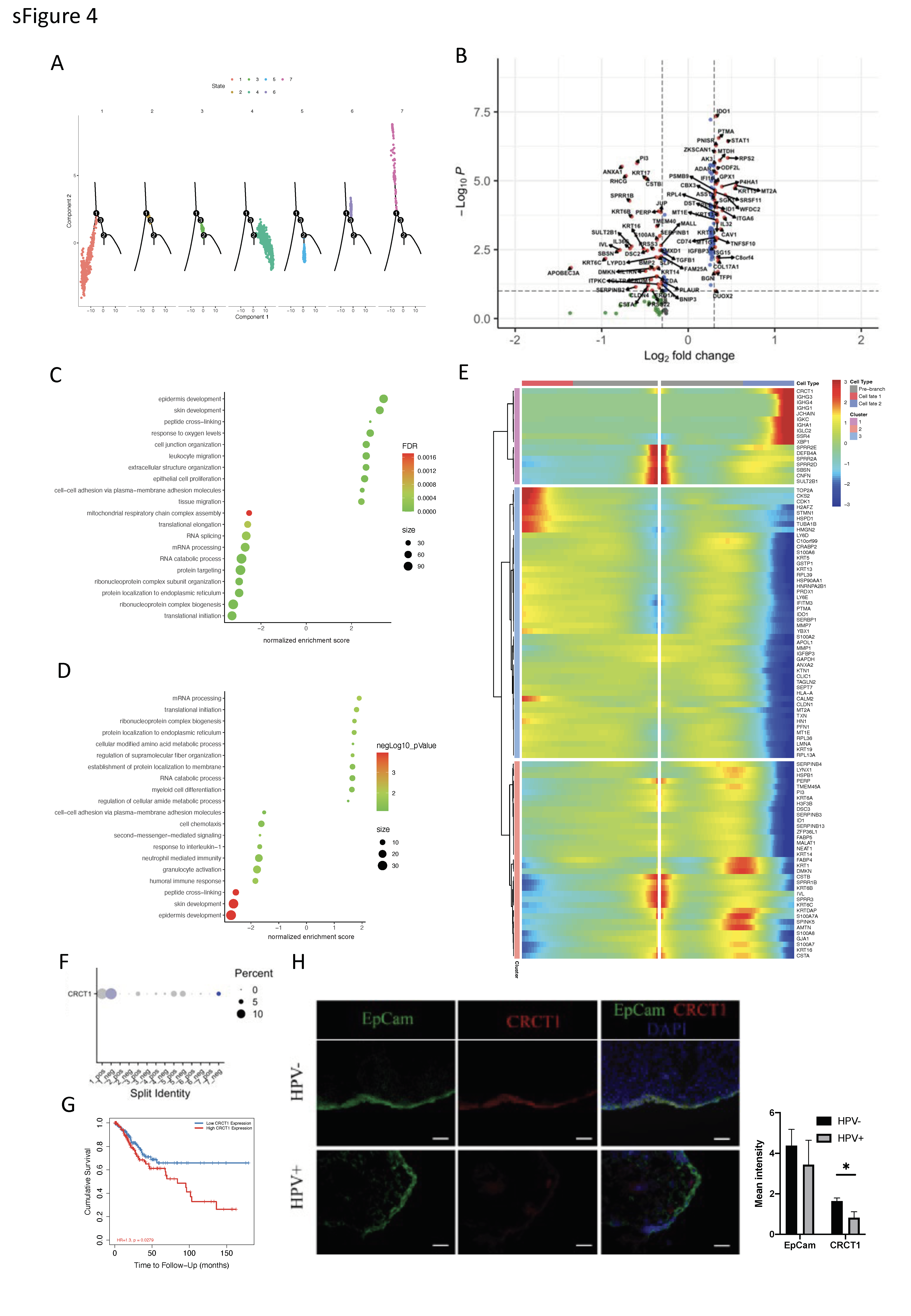

Supplement: Supplementary Figure 4 — Trajectory analysis of HPV infected cervical cancer cells. (A) Trajectory analysis of HPV- (n) and HPV+ (p) tumor cells in different states. States 1 and 7 refer to the beginning and the end of the development. (B) Volcano plots demonstrating the expression patterns and levels of the genes in the HPV+ cells of State 1. (C) The enriched gene ontology of GSEA GO analysis for cancer stemness (State 1)-correlated gene signatures. (D) The enriched gene ontology of GSEA GO analysis for HPV+ State1 cell-correlated gene signatures. (E) BEAM (Branched expression analysis modelling) was used to find the genes that were regulated in a branch-dependent manner. A heatmap showing the temporal differential genes with the top 100 extracted. The horizontal axis of the heatmap refers to the proposed time. The genes shown are those that are highly expressed in the indicated clusters at the focus of the proposed time sort. (F) A dot plot showing the expression of CRCT1 in the seven states of HPV+ or HPV- tumor cells. (G) Kaplan-Meier analysis of overall survival according to the gene expression of CRCT1 in CSCC patients from the TCGA dataset. (H) Representative immunostained photomicrographs of CRCT1 in the tissues obtained from HPV- and HPV+ patients. EpCam staining refers to the squamous cell carcinoma. Scale bar indicates 50μm. Bar graphs depicted the mean fluorescent intensity of the indicated staining. * indicates p<0.05 of six samples. [file Image_4.tiff]

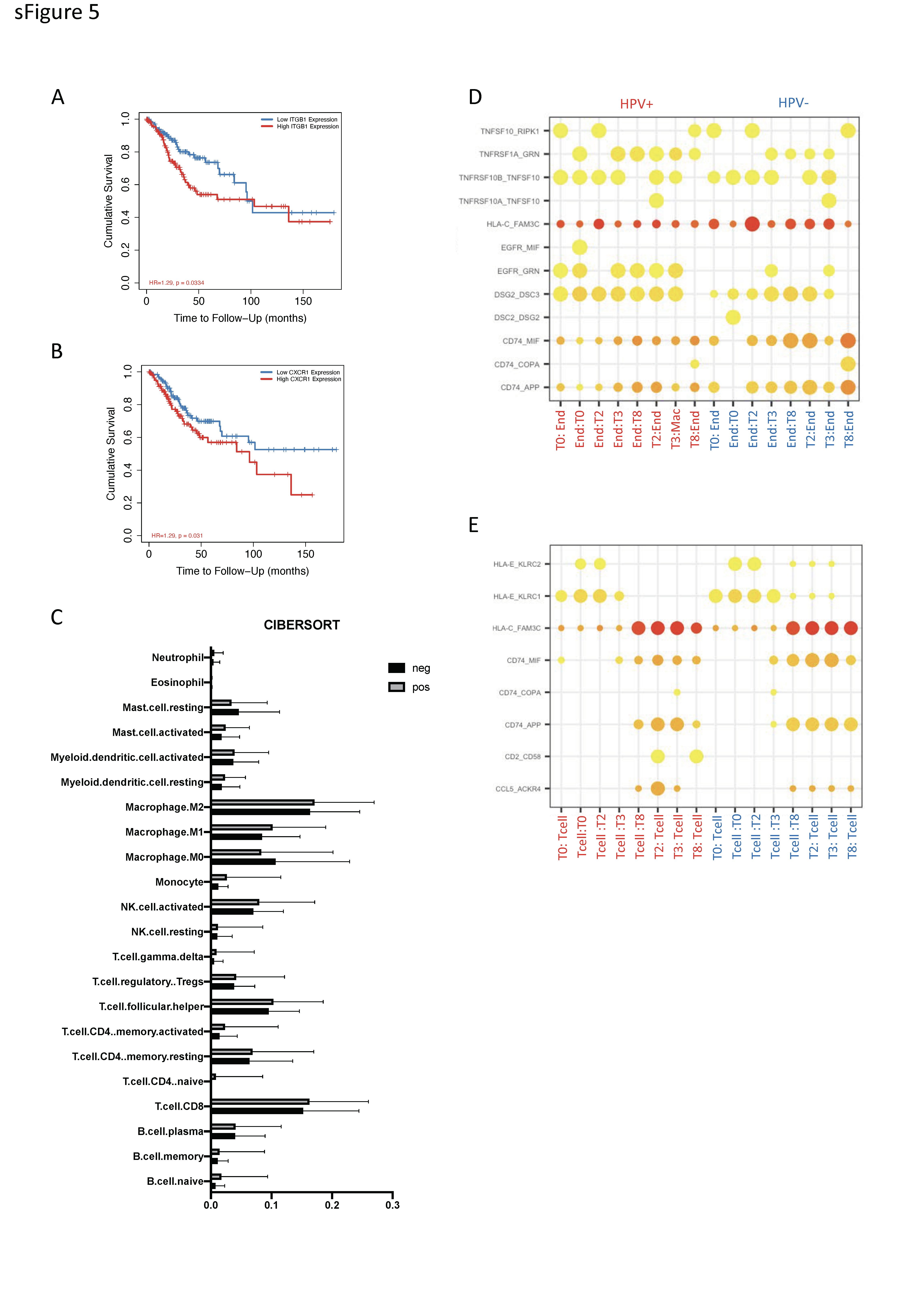

Supplement: Supplementary Figure 5 — Cell-cell communication between immune cells and HPV-infected tumor cells.Kaplan-Meier survival analysis of the HPV- tumor cells using the interaction genes (A) ITGB1 and (B) CXCR1 in the CSCC patients. (C) A bar graph showing the CIBERSORT estimated infiltration of immune cell subsets of samples from HPV+ and HPV- CSCC patients. Dot plots showing the most significant interactions (mean>1) of endothelial cells (D) and T cells (E) with either HPV+ or HPV- tumor cells and the significance of their relationships. The horizontal coordinates are cell type interactions and the vertical coordinates are protein interactions, with the larger dots indicating smaller p-values and the colours representing the average expression. [file Image_5.tiff]

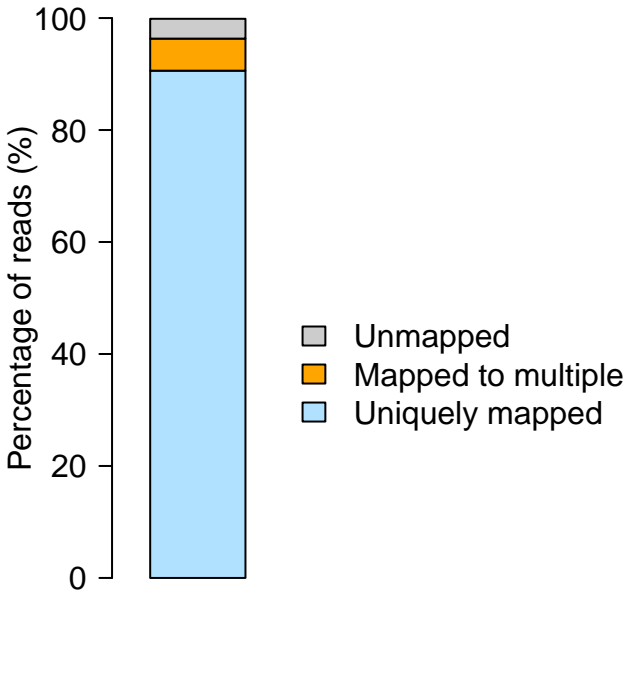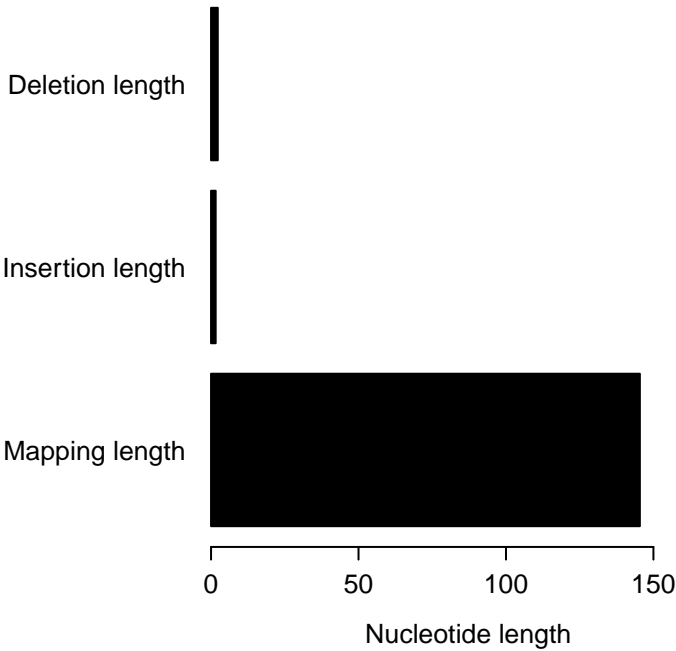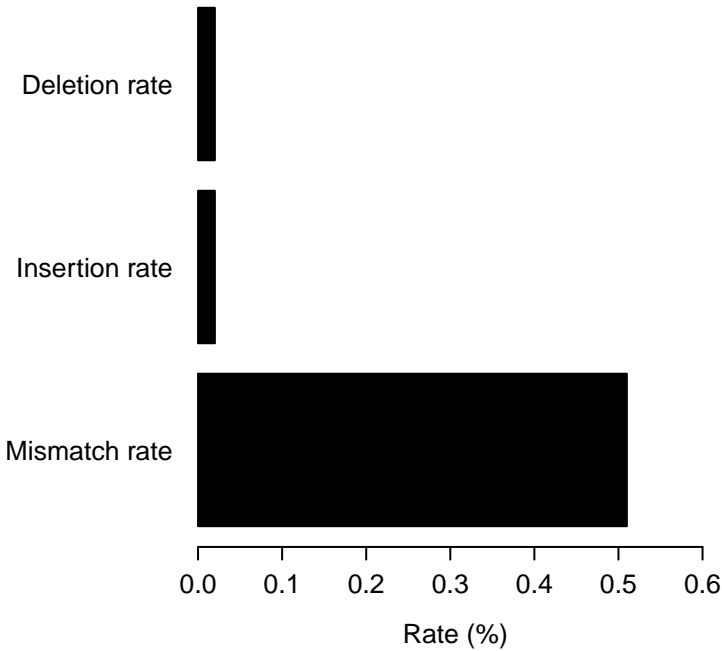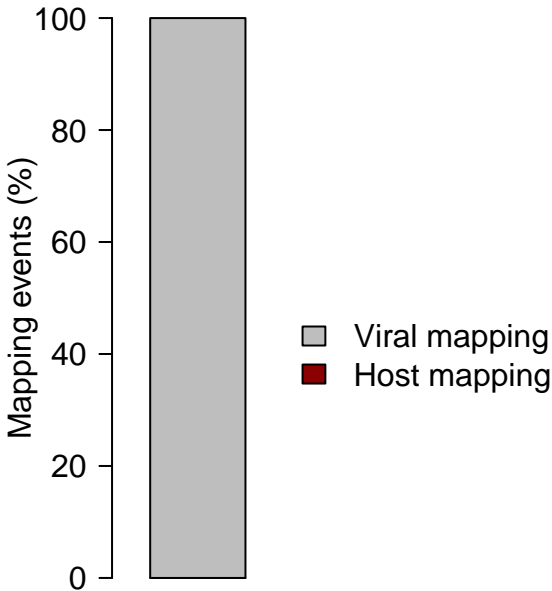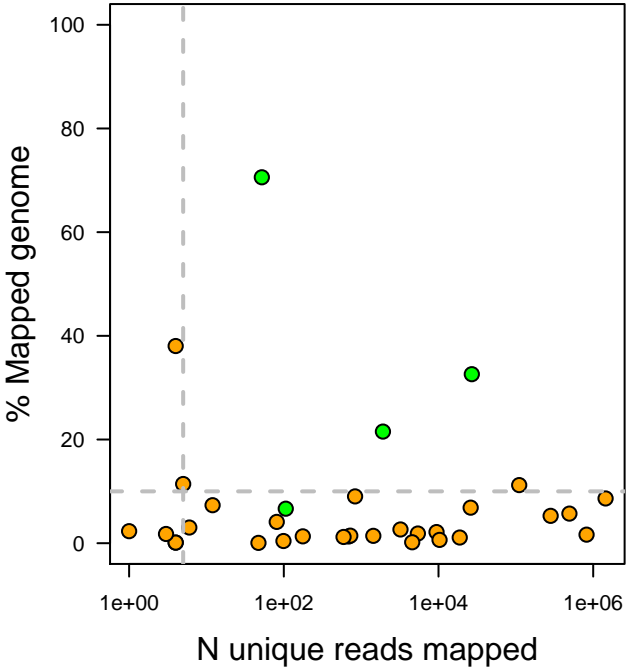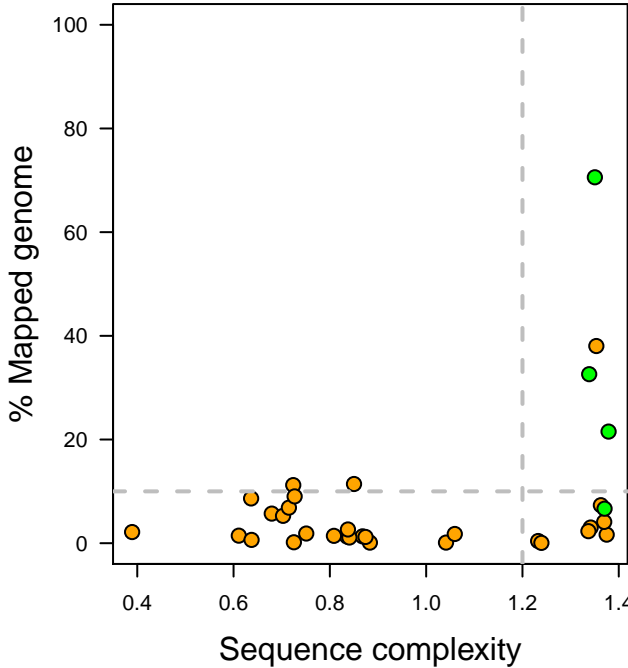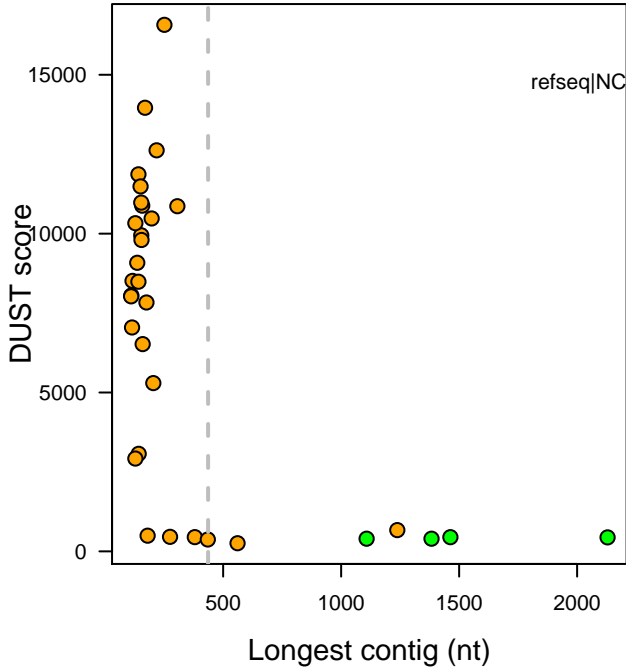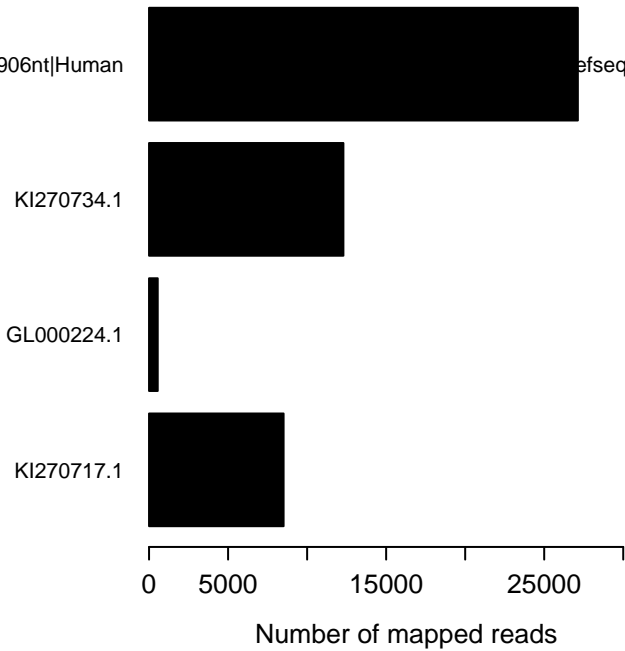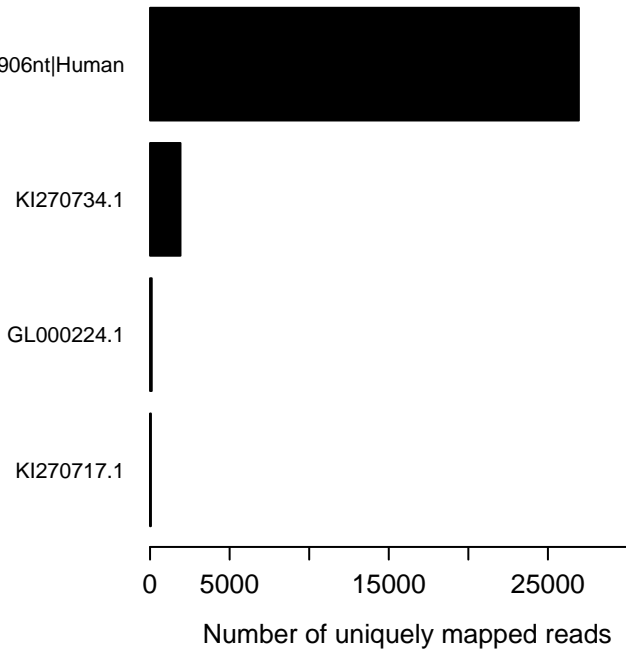

Supplement: Supplementary file 7 [file DataSheet_2.pdf]
